# From "Lemon" and "Banana" Signs to Clinical Management: A Case Report of a Mid-Trimester Chiari II Malformation Diagnosis

**DOI:** 10.7759/cureus.117231

**Published:** 2026-09-30

**Authors:** Ajay Khadka, Pratik Regmi, K C Saroj Raj

**Affiliations:** 1 Department of Radiology, Shree Birendra Hospital, Nepalese Army Institute of Health Sciences, Kathmandu, NPL

**Keywords:** chiari malformation ii, genetic counseling, lemon and banana signs, neural tube defects, prenatal ultrasonography

## Abstract

Chiari II malformation is a significant structural anomaly of the posterior fossa, characterized by the downward herniation of the cerebellum and brainstem, and is frequently associated with open neural tube defects. Early prenatal detection is critical for multidisciplinary clinical management and parental counselling.

This report describes the prenatal diagnosis of a Chiari II malformation in an 18-week gestation fetus. Routine neurosonography revealed the classic sonographic markers of the "lemon sign," which is frontal bone scalloping, and the "banana sign," which is abnormal cerebellar morphology in the presence of an open lumbosacral myelomeningocele. The identification of these markers allowed for a prompt and comprehensive clinical evaluation and informed reproductive decision-making.

The lemon and banana signs remain highly reliable sonographic markers for identifying Chiari II malformation during the mid-trimester anomaly scan. Systematic screening protocols are essential for the accurate detection of these findings.

## Introduction

Chiari II malformation is a complex developmental abnormality characterized by caudal displacement of the posterior fossa contents, including the cerebellum and brainstem, and is strongly associated with open spinal dysraphism, most commonly myelomeningocele [1]. The condition is uncommon in the general population, with published estimates of approximately 0.5-1 per 1,000 births, although the true frequency is difficult to define because of differences in ascertainment, prenatal diagnosis, pregnancy loss, and preventive folate supplementation [2].

The mid-trimester anomaly scan provides an important opportunity to identify open spina bifida through both direct assessment of the spine and characteristic intracranial findings. The "lemon sign," referring to scalloping of the frontal bones, and the "banana sign," referring to the curved or crescentic appearance of the cerebellum with effacement of the cisterna magna, are established sonographic markers of open spina bifida and Chiari II malformation [3,4]. In a classic series of 130 fetuses with open spina bifida, the lemon sign was present in 98% of fetuses at or before 24 weeks, while cerebellar abnormalities were present in 95%; at this gestational age, the banana configuration was the predominant cerebellar abnormality [3].

These findings are highly informative but should be interpreted in conjunction with a systematic examination of the fetal spine and brain. Open and closed forms of spina bifida may have different cranial appearances, and additional intracranial abnormalities--including ventriculomegaly, altered posterior fossa configuration, and other developmental abnormalities--may occur in fetuses with myelomeningocele [5,6]. The lemon sign is also not completely specific and may occasionally be seen transiently in otherwise normal fetuses or in other circumstances; therefore, it should not be interpreted in isolation [6].

Although some open spina bifida lesions may be suspected during the first trimester, the classic lemon and banana signs are primarily recognized during the second trimester, and the sensitivity and appearance of these markers vary with gestational age [7]. Fetal MRI can provide additional anatomical information when ultrasound is limited or when detailed assessment is required for fetal therapy planning, but it was not performed in the present case [6].

This case report describes the prenatal diagnosis of Chiari II malformation at 18 weeks of gestation, emphasizing the characteristic ultrasound findings, the associated spinal defect, and the subsequent clinical management and counselling pathway.

## Case presentation

A 34-year-old woman presented for a routine mid-trimester anomaly scan at 18 weeks of gestation. The pregnancy was achieved by spontaneous conception, and the first trimester was reported as uncomplicated. There was no significant family history of neural tube defects or known exposure to teratogens. There was no history of a previous pregnancy affected by a similar neural tube defect.

During detailed ultrasound assessment at 18 weeks, fetal biometry was consistent with gestational age. Neurosonography demonstrated characteristic features of Chiari II malformation. In the transverse plane of the fetal cranium, there was concave scalloping of the frontal bones, consistent with the "lemon sign." Assessment of the posterior fossa demonstrated an abnormal crescentic configuration of the cerebellum with obliteration of the cisterna magna, consistent with the "banana sign." Mild ventriculomegaly was also observed (Figure 1).

**Figure 1:**
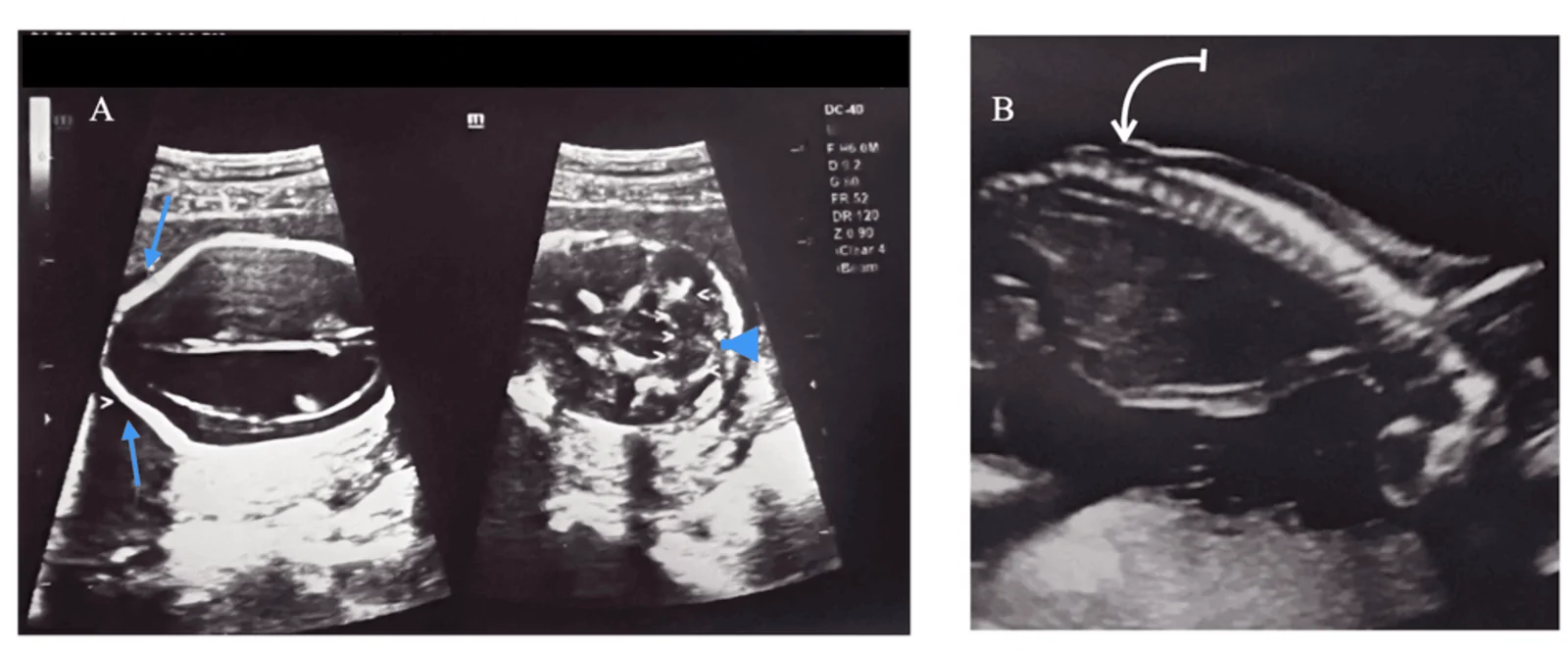
Grey-scale ultrasound. (A) Representative transverse view of the fetal cranium showing bilateral frontal bone scalloping consistent with the "lemon sign" (straight arrows) and representative transverse view of the fetal posterior fossa showing altered cerebellar morphology consistent with the "banana sign" (arrowhead). (B) Longitudinal view of the fetal spine showing a cystic area adjacent and superficial to the lumbosacral spine, consistent with myelomeningocele (curved arrow).

Systematic examination of the fetal spine demonstrated a lumbosacral myelomeningocele. The combination of the characteristic cranial findings and the open lumbosacral spinal defect supported the prenatal diagnosis of Chiari II malformation associated with myelomeningocele.

Fetal MRI was not performed in this case. Genetic testing was also not performed. Following the ultrasound findings, the patient was referred to a tertiary center for maternal-fetal medicine evaluation and genetic counselling. After multidisciplinary discussion regarding the diagnosis, prognosis, and potential long-term clinical implications, the patient and her family opted for pregnancy termination. The procedure was performed by induction of labor and resulted in spontaneous vaginal delivery. Post-delivery clinical examination of the fetus confirmed the presence of the lumbosacral myelomeningocele (Figure 2).

**Figure 2:**
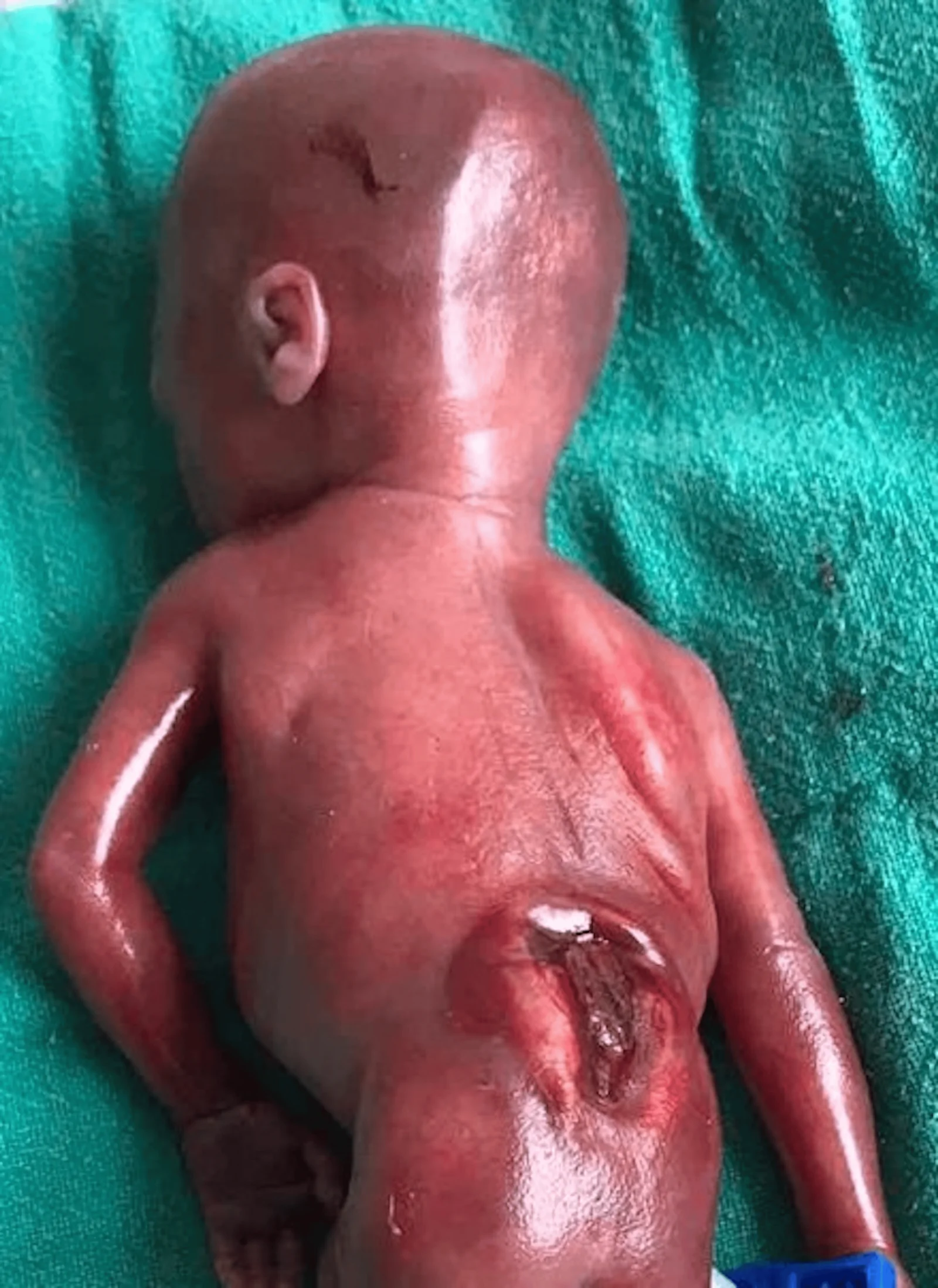
Post-delivery dorsal view of the lumbosacral region demonstrating a myelomeningocele with exposed neural tissue and surrounding erythema.

## Discussion

The prenatal diagnosis of Chiari II malformation is closely linked to the recognition of open spinal dysraphism and its characteristic intracranial manifestations. The underlying abnormality involves caudal displacement of posterior fossa structures and is strongly associated with myelomeningocele [1,2]. In the present case, the simultaneous identification of the lemon sign, banana sign, mild ventriculomegaly, and lumbosacral myelomeningocele at 18 weeks provided a coherent sonographic pattern supporting the diagnosis.

The diagnostic value of the lemon and banana signs is influenced by gestational age. Van den Hof et al. reported the lemon sign in 98% of fetuses with open spina bifida at or before 24 weeks, while cerebellar abnormalities were present in 95%; the banana sign was the predominant cerebellar appearance in fetuses at or before 24 weeks [3]. A subsequent systematic review of second-trimester fetuses with myelomeningocele reported wide ranges in the frequency of individual cranial findings, reflecting differences in gestational age, study populations, and definitions. The banana sign was reported in 50-100% and the lemon sign in 53-100% of affected fetuses across the included studies [4]. These data support the usefulness of the signs while also demonstrating that their absence does not necessarily exclude an underlying spinal defect.

The differential interpretation of fetal cranial and posterior fossa abnormalities is important. The lemon sign is not entirely specific for open neural tube defects and may occur transiently in a small proportion of structurally normal fetuses [6]. Conversely, closed spina bifida generally lacks the characteristic cranial alterations associated with open defects. In a study of 66 prenatally diagnosed cases of spina bifida, 93% of followed cases were open and 7% were closed; the open lesions were associated with typical cranial alterations including the lemon and banana signs, whereas the cranial anatomy was unremarkable in the closed cases [5]. Therefore, recognition of these signs should prompt a careful, systematic examination of the fetal spine rather than reliance on an isolated cranial marker.

Other intracranial abnormalities may accompany Chiari II malformation and myelomeningocele. A systematic review identified ventriculomegaly, small head measurements, abnormal posterior fossa configuration, tectal abnormalities, and other cerebral developmental abnormalities among reported second-trimester ultrasound findings [4,8]. More recent fetal MRI research has also demonstrated developmental differences involving the diencephalon, ventricular system, and proliferative zones in fetuses with Chiari II malformation [2]. These observations emphasize that Chiari II is a broader developmental disorder rather than an isolated posterior fossa abnormality.

Evaluation of the first trimester provides important context regarding the timeline of developmental expression. Early sonographic detection of open spina bifida is possible, and several first-trimester markers have been described; however, the classic lemon and banana signs are principally established as second-trimester markers, and their appearance changes with gestational age [7]. In the present case, the pregnancy was reported as uncomplicated during the first trimester, but no specific first-trimester sonographic marker was documented. Consequently, it cannot be definitively established whether first-trimester sonographic parameters were entirely normal.

Genetic testing was not performed in this case. Neural tube defects are generally considered multifactorial conditions involving interactions among genetic, nutritional, and environmental factors rather than a single causative genetic abnormality [9,10]. The absence of genetic testing limits the ability to comment on a specific chromosomal or molecular etiology in this fetus and is an important limitation of the case.

There was no history of a previous pregnancy affected by a similar neural tube defect. This information is relevant during counselling because recurrence risk and preventive strategies are important components of management of pregnancies affected by neural tube defects [10]. In the present case, counselling was undertaken at a tertiary center after identification of the fetal abnormalities, and the patient and her family subsequently opted for termination of pregnancy.

Fetal MRI was not performed. Although MRI can provide complementary anatomical information when ultrasound is technically limited or when detailed assessment is required for fetal intervention planning, ultrasound remains the principal imaging modality for routine prenatal diagnosis of open spina bifida and Chiari II malformation [6]. In this case, the ultrasound findings were sufficiently characteristic and were subsequently supported by post-delivery clinical examination.

The principal strength of this report is the clear demonstration of the classic sonographic constellation of Chiari II malformation at 18 weeks, including the lemon and banana signs and an associated lumbosacral myelomeningocele, together with documentation of the subsequent multidisciplinary management pathway. The main limitations are the single-case design, absence of fetal MRI, and absence of genetic testing. The report therefore cannot provide population-level estimates of diagnostic accuracy, frequency, or prognosis. Its value is primarily educational, highlighting the importance of systematic neurosonography and spinal assessment during the mid-trimester anomaly scan.

## Conclusions

Chiari II malformation should be considered when characteristic intracranial sonographic findings, particularly the lemon and banana signs, are identified during the mid-trimester anomaly scan. In this case, the combination of these findings with mild ventriculomegaly and a lumbosacral myelomeningocele supported the prenatal diagnosis at 18 weeks of gestation. These markers should be interpreted as part of a systematic assessment of the fetal brain and spine rather than in isolation. Early recognition allows timely referral for multidisciplinary evaluation, prognostic counselling, and discussion of available management options. As illustrated by this case, careful neurosonography remains central to the prenatal detection and characterization of Chiari II malformation associated with open neural tube defects.
